# Comparison of two different double-plate fixation methods with olecranon osteotomy for intercondylar fractures of the distal humeri of young adults

**DOI:** 10.3892/etm.2013.1102

**Published:** 2013-05-08

**Authors:** DASHENG TIAN, JUEHUA JING, JUN QIAN, JIANMING LI

**Affiliations:** 1 Department of Orthopedics, Qilu Hospital Affiliated to Shandong University, Jinan, Shandong 250012;; 2 Department of Orthopedics, Second Hospital Affiliated to Anhui Medical University, Hefei, Anhui 230601, P.R. China

**Keywords:** intercondylar fracture, distal humerus, double plate, internal fixation

## Abstract

Although several studies have demonstrated good results with open reduction and internal fixation of intercondylar fractures of the distal humerus, few have specifically addressed the results of such surgical fixation in young adults. The purpose of this study was to compare the clinical outcomes in patients with intercondylar fractures of the distal humerus treated using two different double-plating methods. Twenty-five patients with distal humeral fractures classified as type C according to the Association for Osteosynthesis/Association for the Study of Internal Fixation (AO/ASIF) classification system, who were admitted to the Second Hospital Affiliated to Anhui Medical University (Hefei, China) from October 2008 to October 2011, were included in the study. The patients were treated with two different double-plate fixation and olecranon osteotomy methods. Thirteen patients were treated by perpendicular plating (group I) and twelve patients by Y-shaped double-plating in the coronal plane (group II). All the patients were followed up for 12–38 months, with an average of 19.2±7.1 months in group I and 18.3±4.0 months in group II. All the osteotomies and fractures had healed by the final follow-up. Complications developed in 4 patients in group I and 3 patients in group II. According to the Mayo Elbow Performance Scores (MEPS), 84.6% of patients in group I and 83.3% in group II had excellent or good scores. No significant differences were identified between the clinical outcomes of the two plating methods. The olecranon osteotomy approach with double-plate fixation is a good choice for the surgical treatment of type C intercondylar fractures in young adult distal humeri. The two plating methods provide solid fixation, permit early rehabilitation and result in satisfactory clinical outcomes.

## Introduction

Comminuted intercondylar fractures of the distal humerus in young adults are difficult to treat due to the complex anatomy of the elbow, small fracture fragments and the limited amount of subchondral bone (1,2). These fractures in young adults often occur as a result of high-energy trauma and usually require early operative treatment with accurate anatomic reduction of the joint fragments and stable fixation to provide early mobilization and satisfactory results (3,4). Open reduction and internal fixation using plates have demonstrated satisfactory clinical outcomes for the treatment of intercondylar fractures of the distal humerus and various plating methods have been described to achieve firm stabilization (5–8). One study demonstrated that double-plate fixation provides more stable fixation than other methods (9). However, controversy remains concerning plate positions in terms of providing optimal stability for intercondylar distal humerus fractures. The widely used double-plate fixation methods involve: i) placing plates parallel to each other in the sagittal plane, with two plates on each supracondylar ridge; ii) placing plates perpendicular to each other, with one on the medial supracondylar ridge and the other on the lateral posterior; and iii) placing plates in a Y shape in the coronal plane, with two plates on the medial and lateral posterior surfaces of the distal humerus. Although a number of studies have compared the first two methods in terms of clinical outcomes or biomechanism (10–12), few studies have compared the clinical outcomes of the last two methods when used to treat intercondylar fractures of young adult distal humeri. The purpose of the present study was to compare the clinical outcomes and complications of perpendicular and Y-shaped double-plating in young adults with intercondylar fractures of the distal humerus.

## Subjects and methods

### Subjects

From October 2008 to October 2011, 29 consecutive patients with intercondylar fractures of the distal humerus were treated with open reduction and double-plate fixation in the Second Hospital Affiliated to Anhui Medical University (Hefei, China). Four patients were lost to follow-up and the remaining 25 patients were followed up for a minimum of 12 months. Patients included in this study were selected based on the following criteria: distal humeral fractures classified as type C according to the Association for Osteosynthesis/Association for the Study of Internal Fixation (AO/ASIF) classification system (13), and a minimum follow-up after surgery of 12 months. Exclusion criteria were: i) suspicion of primary or metastatic tumors with a pathological fracture and ii) age <18 or >60 years.

**Figure 1.**
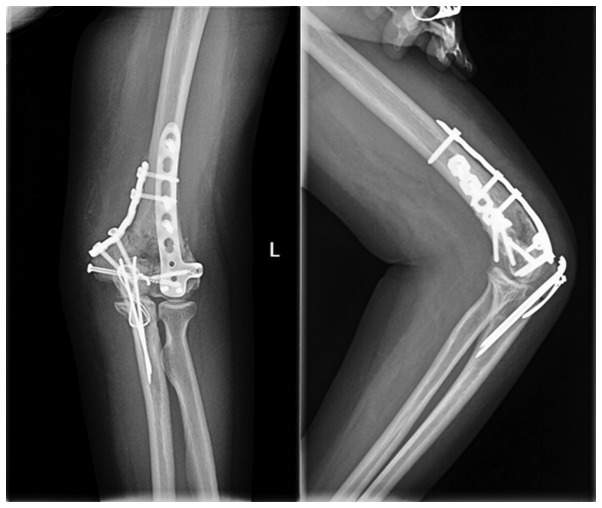
Postoperative radiographs following perpendicular plating fixation for distal humerus fractures.

**Figure 2.**
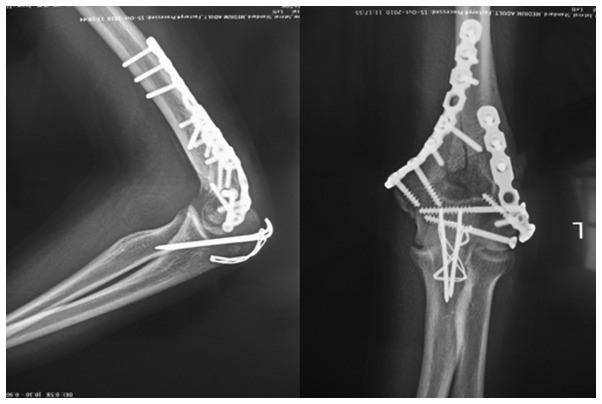
Postoperative radiographs following Y-shaped plating fixation for distal humerus fractures.

The 25 patients were randomly assigned to two groups based on plate position. Thirteen patients were treated using two orthogonal plates (group I): one plate was placed along the medial supracondylar ridge and the other placed postero-laterally, with the plates approximately perpendicular to each other (Fig. 1). Twelve patients were treated using Y-shaped double-plating (group II): plates were placed in a Y shape in the coronal plane, with the two plates on the medial and lateral posterior surfaces of the distal humerus (Fig. 2).

Of the 25 patients, there were 15 males and 10 females (8 males in group I and 7 males in group II; Table I). The mean age at the time of injury was 39.1±12.4 years (range, 19–55 years) in group I and 38.8±12.5 years (range, 18–56 years) in group II. The average time between injury and internal fixation was 6.7±2.9 days in group I and 7.4±2.7 days in group II. According to the AO/ASIF classification system, two fractures were type C1, six type C2 and the remaining five type C3 in group I. Three of the patients in group I had compound injuries of Gustilo type 1 (one patient) or type 2 (two patients) and three patients presented clinical signs of ulnar nerve injury. Among the patients in group II, three were type C1, five were type C2 and the remaining four were type C3. One patient presented compound clinical signs of ulnar nerve injury. No significant differences were observed in terms of demographic data and operative procedures between the patients in the two groups. This study was conducted in accordance with the Declaration of Helsinki and with approval from the Ethics Committee of Qilu Hospital Affiliated to Shandong University. Written informed consent was obtained from all participants.

Pre-contoured anatomical plates and 3.5-mm reconstruction plates were used in this study and all plates were made of titanium. All but two of the patients underwent surgery within 10 days of injury. The surgery of one patient with compound injuries of Gustilo type 2 in group I was delayed until 14 days after injury, and that of one patient in group II with compound craniocerebral trauma was delayed until 12 days after injury. For all patients, computed tomography (CT) was performed preoperatively to identify comminution and to locate fracture fragments accurately, in addition to the conventional two- plane radiography.

All 25 patients included in this study followed the same postoperative rehabilitation protocol. Early controlled passive mobilization was started 48 h postoperatively after the removal of the drainage and a long arm cast was placed with the elbow in 80° flexion for 2 weeks during the interval of mobilization. All patients received celecoxib postoperatively (200 mg every 12 h) for 2 weeks in order to prevent heterotopic ossification and to ease pain. Clinical and radiological evaluations were performed regularly at 3 days, 1 month, 3 months, 6 months and 1 year, then at 6-month intervals. Standard anteroposterior and lateral radiographs were obtained to assess fixation conditions and determine the incidences of nonunion, metal failure and heterotopic ossification. Clinical evaluations consisted of recording the incidences of complications and determining Mayo Elbow Performance Scores (MEPS; Table II) (14).

### Surgical procedures

All surgery was performed under general anesthesia. With the patient in a supine position, a tourniquet was applied high up on the arm and the shoulder and elbow were flexed 90°. A straight posterior approach with slight radial deviation over the olecranon was used; the ulnar nerve was routinely identified and mobilized ≥6 cm proximal and distal to the cubital tunnel. Every effort was made to avoid nerve injury during surgery and anterior subcutaneous transposition of the ulnar nerve was usually performed to prevent ulnar nerve tension or impingement over the plate following surgery. Following blunt dissection of the triceps at the medial and lateral intermuscular septum, medial visualization of the olecranon joint was performed to identify the bare area. Prior to osteotomy, two K-wire holes for refixation were drilled from the olecranon tip to the ulna coracoid process and holes were drilled in the ulna for subsequent tension band wiring. Using a thin oscillating saw, a V-shaped olecranon osteotomy was created ∼2.5 cm distal to the olecranon tip. The osteotomy was completed by fracturing the last third of the ulna, creating an irregular osteochondral fracture line for improved interdigitation and facilitated reduction. Through a V-shaped olecranon osteotomy, the distal humerus articular joint was exposed. With the articular fragment of the distal block in view, the trochlea was reduced first. Large fragments were fixed with transverse cannulated screws and small fragments with 1-mm K-wires. Special attention was made to restore the normal length and width of the trochlea for type C3 fractures. Then, the remaining fragments were reducted and K-wires were passed for temporary fixation. Definitive fixation of the articular fragments to the bone columns was based in the use of strategically placed osteosynthesis, preserving as much soft-tissue attachment to bone as possible. In group I, bone columns were reduced and stabilized with two plates: usually a 3.5-mm reconstruction plate or a pre-contoured anatomical plate placed along the medial supracondylar ridge and another 3.5-mm reconstruction plate or a pre-contoured anatomical plate in the posterior aspect of the lateral column. Two plates were contoured to fit the reduced distal humeral column during surgery. In group II, two 3.5-mm reconstruction plates were applied on the medial and lateral posterior surfaces of the distal humerus, in a Y shape in the coronal plane. Fracture lines in the coronal plane and small articular fragments were stabilized using 2.7-mm cannulated screws. Following definitive fixation, all K-wires were removed as far as possible. The olecranon osteotomy was fixed at the end with two K-wires and tension band wiring in all cases.

### Statistical analysis

Statistical analyses were performed with Statistical Package for Social Science (SPSS version 16.0; SPSS, Inc., Chicago, IL, USA). All continuous variable values are expressed as mean ± standard deviation (SD). A two-sample t-test was used for continuous variables (range of motion, flexion, extension, MEPS, age and time to surgery) and Fisher’s exact test for dichotomous variables to compare demographic data (rate of excellent and good MEPS, fracture type, male to female ratio and right to left ratio). The level of statistical significance was defined as P=0.05.

## Results

All patients were followed up for 12 to 38 months with an average of 19.2±7.1 months in group I and 18.3±4.0 months in group II. All the osteotomies and fractures healed radiographically without a step-off at the articular margin >2 mm or an angular deformity >10° at the last follow-up. Nineteen screws were used in group I and 22 in group II. No complications associated with the olecranon osteotomies were encountered. The majority of patients achieved bony union at 6 months (fourth radiological evaluation) postoperatively and only one patient had delayed bony union until 8 months after surgery in group II. No screw loosening, plate breakage or deep infection was observed. Mild occasional pain was reported by six patients in group I and five in group II, and none of the patients in either group reported severe or constant pain.

The arc of motion averaged 106.2±22.0° postoperatively with a mean elbow flexion of 120.8±12.6° (range, 100–135°) and extension of 14.6±10.5° (range 0–40°) in group I (Table III). In group II, the arc of motion was 105.0±21.7° with a mean elbow flexion of 119.6±13.6° (range, 95–135°) and extension of 14.6±10.8° (range 0–40°) postoperatively. The average MEPS was 89.6±11.8° (range, 70–100°) in group I and 90.0±12.3° (range, 70–100°) in group II. According to MEPS, 84.6% of patients in group I and 83.3% in group II had excellent or good scores. No statistically significant inter-group differences were evident in terms of clinical outcomes.

Complications developed in four patients in group I and three patients in group II. According to the scale of Knirk and Jupiter (15) for the assessment of post-traumatic arthritis, two elbows in group I and one in group II were grade 1. One patient in group II (a 22-year-old male with a C3 fracture) who had grade I heterotopic ossification, per the Hastings and Graham classification (16), presented no functional impairment. Transient ulnar nerve neuropathy developed in one patient in group I (a 32-year-old male with a C3 fracture). Ulnar nerve symptoms in all patients, including four patients suffering from the symptoms preoperatively, had subsided completely 6 months after surgery. Two patients had an unfavourable arc of motion (60 and 65°) in the final follow-up. One patient in group I (a 50-year-old female with a C3 fracture) was mentally handicapped and one patient in group II (a 56-year-old female with a C3 fracture) had surgical neck fractures of the humerus.

## Discussion

The aim of treatment for distal humerus fractures is to make the elbow stable and painless with satisfactory function. This requires anatomic reconstruction of the articular surface, restitution of the overall geometry of the distal humerus and stable fixation of the fracture fragments to allow early and full rehabilitation. Although these goals are now widely accepted by the orthopedic community (8,11,12,17,18), they may be technically difficult to achieve, particularly in the presence of substantial comminution.

Within the last several years, a two-column theory of the anatomy of the distal humerus has been advocated whereby the coronal plane of the distal humerus is considered to be in the shape of a triangle, with the coronoid fossa and olecranon fossa accounting for the majority of the central area and the medial and lateral condyles forming two strong columns by proximal extension (8,19). Fixation of the distal humerus must not only restore the capitulum-trochlea joint, but also the integrity of the medial and lateral columns. Despite controversies concerning the appropriate treatment of distal humerus fractures, double-plate fixation has been widely reported to produce satisfactory clinical outcomes, even in patients with complex intra-articular fractures (7,20). A number of studies have compared the clinical outcomes of parallel and perpendicular plating systems for distal humerus fractures (12,21). Additionally, another study reported the clinical outcomes of perpendicular or Y-shaped double-plating systems for distal humerus fractures (22). Fewer studies have compared the clinical outcomes of the perpendicular and Y-shaped double-plating systems for distal humerus fractures. In the present study, we compared the clinical outcomes of the perpendicular and Y-shaped double-plating methods when applied to type C distal humerus fractures in young adults. Postoperative analyses revealed no significant differences between the two plating modalities in terms of arc of flexion, function, rate of excellent and good MEPS and other clinical results. No screw loosening or plate breakage was observed in any of our patients during the follow-up. Zero incidence of fixation loss indicates that the two methods of placing plates result in an equally stable fixation. The following two points are important: i) the anatomic reconstruction of the intercondylar fractures and use of transverse cannulated screws allows improvement of the fracture from type C to type A; and ii) the plate should be made to fit the shape of the distal humerus and the screws should be tightened one at a time, as far as possible.

Postoperative complication rates of up to 48% have been reported for type C distal humerus fractures (19,23,24). In the present study, one patient in group II suffered from transient ulnar nerve palsy following internal fixation of the distal humerus fracture; however, no patients suffered permanent nerve dysfunction. This may be attributed to anterior transposition of the ulnar nerve. The reported prevalence of heterotopic ossification following surgical treatment of distal humerus fractures ranges from 4 to 49%, although no functional deficit was involved in the majority of cases (25). In the present study, one patient with heterotopic ossification was encountered in group II. This low incidence (4%) of heterotopic ossification may be attributed to the routine use of celecoxib postoperatively. Therefore, we suggest that patients take a nonsteroidal drug routinely.

Reported total ranges of elbow motion vary from 103 to 112° following the double-plating fixation of type C distal humerus fractures, regardless of the plate position (5,26). In the present study, 21 of 25 patients (84%) obtained good or excellent functional results with a mean range of elbow motion of 105.6±21.4° following double-plate fixation. No significant difference was observed in the total range of elbow motion between the two groups. Two patients suffered from an unfavourable arc of motion (60 and 65°) in the final follow-up. The reason for this was that the patients were unable to cooperate with exercise and physical therapy postoperatively. Early and painless functional exercise postoperatively is key to obtaining a good range of elbow motion. However, there is a contradiction between immobility and early functional exercise. All the patients in the two groups were young adults with no osteoporotic bone. Stable fixation was achieved during surgery in all patients and early functional exercise was allowed postoperatively. We advise the initiation of passive exercise after removal of the drainage tube, with the forearm gravity driving the elbow joint to a continuous passive stretch to maximum activity and the other hand supporting the buckling to maximum activity. The exercise was performed for two periods a day after half an hour of taking celecoxib for 20–30 min per period. Plaster immobilization was used during the intervals of exercise in the first weeks, then exercise intensity was increased and a triangular bandage was used during the intervals of exercise. The ideal state may be achieved at ∼6 weeks. The method was simple with a slow and gentle action, causing only light pain and avoiding further injury of the soft tissue and myositis ossificans by violent passive flexion and stretching of the elbow joint.

The limitations of this study included a relatively small number of patients for determination of clinical superiority related to plate position and a lack of comparison of clinical outcomes according to plate position using locking and unlocking plates.

From a clinical perspective, no significant differences were observed between the perpendicular and Y-shaped double-plating methods for distal humerus fractures in terms of clinical outcomes and complication rates. If appropriately applied with suitable plates, the perpendicular and Y-shaped double-plating methods provide adequate stability and anatomic reconstruction of intercondylar fractures of young adult humeri.

## Figures and Tables

**Table I.** Patient demographics in the two groups.

Variable	Perpendicular plating (group I, n=13)	Y-shaped plating (group II, n=12)	P-value
Age (years)	39±12.4 (19–55)	38.8±12.5 (18–56)	0.953
Male : female	8:5	7:5	1
Right : left	4:9	5:7	0.688
Time to surgery (days)	6.7±2.9 (0–12)	7.4±2.7 (5–14)	0.539
Fracture type (AO)	C1, 2; C2, 6; C3, 5	C1, 3; C2, 5; C3, 4	0.834

Age and time to surgery are expressed as mean ± standard deviation (range). AO, Association for Osteosynthesis classification.

**Table II.** Mayo Elbow Performance Score.

Function	Points	Definition (points)
Pain	45	None (45)
		Mild (30)
		Moderate (15)
		Severe (0)
Motion	20	Arc >100° (20)
		Arc 50–100° (15)
		Arc <50° (5)
Stability	10	Stable (10)
		Moderate instability (5)
		Gross instability (0)
Function	25	Comb hair (5)
		Feed (5)
		Perform hygiene (5)
		Put on shirt (5)
		Put on shoe (5)
Total	100	

Classification: excellent, >90; good, 75–89; fair, 60–74; poor, <60.

**Table III.** Clinical comparison between two different plating methods.

Variable	Perpendicular plating (group I)	Y-shaped plating (group II)	P-value
Range of motion (°)	106.2±22.0	105.0±21.7	0.892
Flexion (°)	120.8±12.6 (100–135)	119.6±13.6 (95–135)	0.821
Extension (°)	14.6±10.5 (0–40)	14.6±10.8 (0–40)	1.00
MEPS (°)	89.6±11.8 (70–100)	90.0±12.3 (70–100)	0.935
Rate of excellent and good scores (%)	84.6	83.3	1.00

Data are presented as mean ± standard deviation (range). MEPS, Mayo Elbow Performance Score.
